# Cefepime neurotoxicity in the intensive care unit: a cause of severe, underappreciated encephalopathy

**DOI:** 10.1186/cc13094

**Published:** 2013-11-07

**Authors:** Jennifer E Fugate, Ejaaz A Kalimullah, Sara E Hocker, Sarah L Clark, Eelco FM Wijdicks, Alejandro A Rabinstein

**Affiliations:** 1 Division of Critical Care Neurology, Mayo Clinic, 200 First Street SW, Mayo W8B, Rochester, MN 55905, USA; 2 Division of Pulmonary and Critical Care Medicine, Mayo Clinic, 200 First Street SW, Mayo W8B, Rochester, MN 55905, USA; 3 Department of Pharmacy, Mayo Clinic, 200 First Street SW, Mayo W8B, Rochester, MN 55905, USA

## Abstract

**Introduction:**

Cefepime, a broad spectrum antibiotic, is commonly prescribed in intensive care units (ICU) and may be an overlooked cause of neurologic symptoms such as encephalopathy, myoclonus, seizures, and coma. We aimed to characterize cefepime neurotoxicity in the ICU.

**Methods:**

We performed a retrospective study of adult ICU patients treated with intravenous cefepime for at least 3 days between January 1, 2009 and December 31, 2011. The primary outcome was the development of cefepime neurotoxicity, with the likelihood of causality ascribed via a modified Delphi method.

**Results:**

This study included 100 patients. The mean age was 65.8 years (± 12.7 years). The median daily average dose of cefepime was 2.5 (IQR 2.0 to 3.5) grams. The median treatment duration was 6 (IQR 4 to 10) days. Renal failure in any form was present in 84 patients. Chronic kidney disease affected 40 patients, and 77 had acute kidney injury. Cefepime neurotoxicity occurred in 15 patients. Of these, seven were considered definite cases, three probable, and five possible. Neurotoxic symptoms included impaired consciousness (n = 13), myoclonus (n = 11), disorientation (n = 6), and nonconvulsive status epilepticus (n = 1). The dose of cefepime was appropriately adjusted for renal clearance in 64 patients (75.3%) without cefepime neurotoxicity and four patients (28.6%) with neurotoxicity (*P* = 0.001). Chronic kidney disease was present in 30 patients (35.3%) without neurotoxicity and in 10 (66.7%) of those with neurotoxicity (*P* = 0.04).

**Conclusions:**

Critically ill patients with chronic kidney disease are particularly susceptible to cefepime neurotoxicity. Myoclonus and impaired consciousness are the predominant clinical manifestations. Neurotoxic symptoms occur more often when the cefepime dose is not adjusted for renal function, but can still occur despite those modifications.

## Introduction

Potent, broad-spectrum antimicrobials are increasingly prescribed in intensive care units (ICUs) because of the proliferation of multidrug-resistant pathogens. Cefepime, a fourth-generation cephalosporin, is one of these antimicrobials commonly prescribed empirically for nosocomial infections. Because cefepime is predominantly renally excreted (85% unchanged), a reduction in renal function confers a proportional increase in the elimination half-life and reduction in total body clearance of cefepime [1]. In patients with renal failure, cefepime can accumulate in both the blood and cerebrospinal fluid and reach toxic concentrations [2]. Consequently, patients with renal failure who are treated with cefepime at relatively high doses are at risk of developing neurological symptoms that include seizures, hallucinations, confusion, myoclonus, and coma [3-12].

The terms ‘encephalopathy’ or ‘delirium’ generically describe cerebral dysfunction of some type, but most of the time they are used to characterize patients with a change in attention, perception and memory. These neurological manifestations are very poorly understood considering their high prevalence in the ICU [13,14]. Given the pervasiveness of confounding causes of encephalopathy (for example infection, postoperative state, electrolyte disturbances, hypoglycemia, uremia, shock, alcohol withdrawal, pain, hypercapnia, hypoxemia), elucidating the cause of ‘altered mental status’ or ‘failure to awaken’ in a patient in the ICU can be challenging. Despite this etiologic uncertainty, the encephalopathy often resolves. However, in persistent cases without readily apparent causation, assessment for potential medication toxicity may prove explanatory. Antibiotics can cause neurologic symptoms, including penicillins, cephalosporins, fluoroquinolones, tetracyclines, sulfonamides, and metronidazole [15]. In our recent experience, we have noted that cefepime neurotoxicity may be a particularly underappreciated phenomenon in ICUs [16].

In this study we aimed to describe the features of a cohort of patients with cefepime neurotoxicity.

## Materials and methods

This was a retrospective observational study conducted at the Mayo Clinic in Rochester, Minnesota. We performed a preliminary search of our pharmacy database for patients who had cefepime profiled on their medication list during hospitalization in an ICU at our institution between January 1, 2009 and December 31, 2011. To identify our cohort of interest, we then entered these results into our electronic Mayo Medical Record Retrieval System and searched the clinical notes for diagnoses of ‘renal failure, kidney injury, renal insufficiency, kidney disease, renal disease, tubular necrosis, end-stage renal disease (ESRD), acute tubular necrosis (ATN), chronic kidney disease (CKD), acute kidney injury (AKI), chronic renal insufficiency (CRI), or acute renal failure (ARF)’ during the same time period. We also searched for the terms ‘encephalopathy, delirium, altered mental status (AMS), confusion, or acute confusional state’. Adult patients who received ≥3 consecutive days of treatment with IV cefepime in the ICU were included.

We reviewed medical records and collected demographics, the duration of cefepime course, mean daily dose of cefepime, serum creatinine and blood urea nitrogen (BUN) concentrations on the first day of cefepime administration, baseline creatinine, whether hemodialysis was required during cefepime administration, and clinical outcome at the time of hospital dismissal. Acute kidney injury was defined as a 1.5-fold increase from baseline creatinine or absolute increase in creatinine by ≥0.3 mg/dL. A patient was considered to have chronic kidney disease if their baseline estimated glomerular filtration rate (eGFR) was ≤60 mL/min/1.73 m^2^ for ≥3 months^.^ Dose adjustments for renal function considered appropriate based on the recommendations in the online Micromedex™ 2.0 database. For example, for a usual dose of 2 grams (g) every 12 hours, if creatinine clearance (CL_Cr_) is 30 to 60 mL/min, an appropriate adjustment is 2 g every 24 hours. If CL_Cr_ is 11 to 29 mL/min, the correct adjustment is 1 g every 24 hours. For patients receiving continuous renal replacement therapy, a dose ≤1 g every 12 hours was considered appropriate, except when prescribed for the indication of febrile neutropenia or severe life-threatening infections, in which case higher doses were considered acceptable. This threshold was determined based on the dosing recommendations from the Mayo Clinic antimicrobial therapy guide, which is developed by experts in our infectious diseases division and is updated every few years based on the prevalence of specific organisms/infections (and resistance rates) in units at our institution. Medical records were reviewed to determine whether there were neurologic symptoms that coincided with the administration of intravenous (IV) cefepime. The likelihood of causality was ascribed via a modified Delphi method; in order for symptoms to be attributed to cefepime neurotoxicity, the following criteria had to be met: 1) neurologic symptoms consisting of encephalopathy, including decreased level of alertness, myoclonus, seizures, or any combination of these, 2) no alternative cause for neurological deterioration, 3) clear temporal relationship between neurologic symptoms and cefepime administration (that is symptoms had to begin following initiation of cefepime, persist or worsen during cefepime administration, and improve or resolve when the medication was discontinued). If symptoms improved spontaneously and there was no change in cefepime dosing, they were not attributed to cefepime. Possible cases were identified by three abstractors (JEF, EAK, SEH). Any possible cases were then reviewed by five co-authors (JEF, EAK, SEH, EFW, AAR) independently. Upon review, when encephalopathy was attributed to cefepime by all five reviewers, the case in question was considered a ‘definite’ case. When four reviewers agreed, it was considered a ‘probable’ case, and when three reviewers agreed, the case was considered ‘possible’.

### Standard protocol approvals, registrations, and patient consents

This study was approved by the Mayo Clinic Institutional Review Board. All patients included in this study had given informed consent to use their medical records for research purposes.

### Statistical analysis

Descriptive summaries were reported as mean ± standard deviation or median and interquartile range (IQR) for continuous variables, with counts and frequencies for categorical variables. Comparisons between subgroups with dichotomous or ordinal variables subgroups were performed with chi-square test or two-sided Fisher’s exact test. For comparisons of continuous variables, we used the Wilcoxon rank sum test. Probability (*P*) values <0.05 were considered statistically significant. We used JMP 9.0, a SAS-based statistical package (SAS Institute, Cary, NC, USA), to analyze the data.

## Results

A total of 100 patients were included. Mean age was 65.8 years (± 12.7 years) and 61 were men. The median daily average dose of cefepime was 2.5 grams (IQR 2.0 to 3.5 g). Indications for antibiotic therapy are shown in Table 1. Patients were hospitalized in the medical ICU (n = 66), surgical ICU (n = 19), trauma ICU (n = 9), cardiovascular ICU (n = 5), or neuroscience ICU (n = 1). The median acute physiology and chronic health evaluation (APACHE) III score - available for 93% - was 85 (IQR 71.5 to 110.5) The median duration of treatment was 6 days (IQR 4 to 10 days). Renal failure in any form was present in 84 patients. Chronic kidney disease affected 40 patients, and 77 had acute kidney injury. Thirty-three patients received renal replacement therapy during the course of cefepime. Of these, 18 underwent continuous veno-venous hemofiltration and 15 were treated with intermittent hemodialysis. The median baseline creatinine of the cohort was 1.0 mg/dL (IQR 0.8 to 1.3 mg/dL). The median creatinine at the start of cefepime therapy was 1.6 mg/dL (1.0 to 2.3 mg/dL), median blood urea nitrogen was 41 mg/dL (IQR 26.3 to 62.5 mg/dL) and median eGFR was 34 mL/min/1.73 m^2^ (IQR 24.3 to 59.5 ml/min/1.73 m^2^). Electroencephalograms (EEGs) were performed during the cefepime course in 17 patients of the entire cohort. Findings included moderate or severe generalized slowing in twelve (70.6%), triphasic waves in eight (47.1%), multifocal sharp waves in five (29.4%), burst-suppression in two (11.8%), non-reactive alpha in one (n = 5.8%), and nonconvulsive status epilepticus (NCSE) in 1 (5.8%).

**Table 1:** Indication for cefepime administration in 100 ICU patients

**Indication**	**n**
Respiratory tract infection	36
Sepsis/bacteremia	23
Empiric	23
Abdominal infection	7
Urinary tract infection	3
Miscellaneous	6
Cellulitis or fasciitis	3
Endocarditis	1
Joint infection (shoulder)	1
Subdural empyema	1

**Figure 1:**
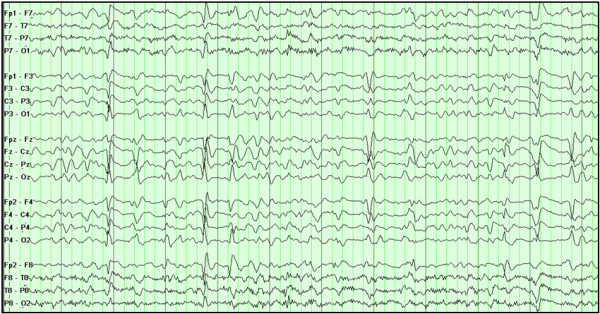
**EEG findings in cefepime neurotoxicity.** Electroencephalogram (longitudinal bipolar montage) of a patient receiving intravenous (IV) cefepime shows diffuse slowing of the background, atypical triphasic waves, and multifocal sharp waves.

Cefepime neurotoxicity occurred in 15 patients. Of these, seven were considered definite cases, three probable, and five possible. The daily doses of cefepime were precisely known for 14 cases (93.3%). The dose had been appropriately adjusted according to renal function in four (28.6%) patients with cefepime neurotoxicity. Neurologic symptoms began at median day 3 (range 1 to 7 days) after cefepime initiation and included depressed level of consciousness (n = 13), myoclonus ( n = 11), disorientation (n = 6), and NCSE (n = 1). Myoclonus was predominant in the facial muscles in six patients and generalized throughout the body in five patients. When predominant in facial muscles, it often involved the periocular muscles, and half of patients had concomitant involvement of one extremity or abdominal muscles. EEGs were performed in nine patients (60%) with cefepime neurotoxicity. The most common findings were diffuse background slowing, triphasic waves, and multifocal sharp waves (example shown in Figure 1). Only one patient had electrographic seizures. Detailed results of EEGs performed in these cases are shown in Table 2.

**Table 2:** Electroencephalogram results of patients with cefepime neurotoxicity

**N**	EEG findings*
2	Atypical triphasic waves
2	Severe diffuse slowing
1	Triphasic waves and multifocal sharp waves
1	Stimulus-induced rhythmic sharp waves over the midline region (SIRPIDs)
1	Multifocal and quasi-periodic sharp waves
1	Multifocal sharp waves
1	Continuous generalized sharp and slow wave discharges (NCSE) and triphasic waves

*All EEGs additionally showed moderate-severe nonspecific diffuse slowing of the background. EEG, electroencephalogram; SIRPIDs, stimulus-induced rhythmic, periodic, or ictal discharges; NCSE, nonconvulsive status epilepticus.

Over two-thirds of patients (n = 72, 69.9%) died during the hospitalization. Of those who died, 19 patients (26%) received cefepime until their death, making it impossible to determine if the encephalopathy may have been related to cefepime administration.

Results comparing clinical characteristics in patients who developed cefepime neurotoxicity and the rest of the cohort are shown in Table 3. Patients who developed cefepime neurotoxicity were less likely to have received an appropriate dose reduction based on renal clearance compared to those who did not develop neurotoxicity (28.6% vs. 75.3%, *P* = 0.001) and more likely to have a history of CKD (66.7% vs. 35.3%, *P* = 0.04).

**Table 3:** Characteristics of 100 ICU patients receiving intravenous (IV) cefepime

	**Cefepime neurotoxicity**	**Rest of cohort**	***P *** **value**
	**n = 15**	**n = 85**	
Age, years, mean	69	66	0.16
Male gender, n (%)	11 (73)	50 (59)	0.39
Acute kidney injury, n (%)	13 (87)	64 (75)	0.51
Chronic kidney disease, n (%)	10 (67)	30 (35)	**0.042**
Hemodialysis, n (%)	4 (27)	28 (33)	0.77
Peak creatinine, median (IQR)	2.8 (1.7-3.1)*	2.3 (1.5-3)	0.36
Nadir eGFR, median (IQR)	22.5 (20.8-34.3)	27.5 (18-45)	0.53
Mean daily cefepime dose, g, median (IQR)	2.5 (1.7-4)*	2.5 (2-3.5)	0.66
Cefepime duration, days, median (IQR)	5 (4.8-7.3)*	7 (4-10)	0.26
Appropriate dose reduction for renal function, n (%)	4 (29)*	64 (75)	**0.001**

*Data available for 14 of the 15 cases of cefepime neurotoxicity. IQR, interquartile range; g, grams.

## Discussion

In this retrospective, single-center study of ICU patients with renal failure, cefepime neurotoxicity was common, particularly in this sample of mostly older patients with pre-existing renal failure. Cefepime neurotoxicity was significantly more frequent in patients without appropriate dose adjustments for renal function compared to those with dose reductions. The more common clinical manifestations of cefepime neurotoxicity included impaired consciousness, encephalopathy, and myoclonus.

Cefepime has received recent attention regarding its potential to cause neurologic complications, and the risk of seizures has been particularly emphasized. In June 2012 the United States Food and Drug Administration released a safety announcement reminding clinicians to adjust the dose of cefepime in patients with renal impairment because of the possibility of seizures (and specifically, of NCSE) [17]. The report focused on seizure activity, an undoubtedly concerning adverse event. There have been nearly 60 cases of cefepime-associated NCSE reported, and ongoing seizure activity played a role in at least one of these patient’s deaths [17]. Periodic sharp waves and triphasic waves are characteristically seen in cefepime neurotoxicity [6]. In one recent study, periodic epileptiform discharges on EEG were five times more frequent in patients receiving cefepime compared to patients receiving meropenem, but overall the prevalence of this finding was still relatively low (1.3%) [18].

While it is imperative that health-care providers are aware of the potential complication of seizures associated with cefepime use, our findings indicate that seizures and NCSE are very uncommon clinical expressions of cefepime neurotoxicity. Rather, the more likely clinical scenario is a patient who develops difficult-to-explain ‘altered mental status’, which may be either a depression in the level of consciousness or confusion or disorientation. Associated myoclonus and renal impairment are potentially suggestive of the diagnosis and should prompt the discontinuation of cefepime in favor of an alternative antibiotic. We have observed patients with renal failure and severe encephalopathy or coma who improved dramatically after the cessation of cefepime. Some of these patients were so seriously affected that discussions about withdrawal of life-sustaining measures had occurred. Stopping a medication is a simple therapeutic trial. Because cefepime neurotoxicity is a reversible (and preventable) cause of severe neurologic symptoms, it is important for clinicians to be aware of this possibility and consider the use of alternative antibiotics in patients with renal impairment.

Decreasing the dose of cefepime in accordance with a patient’s estimated renal function is recommended, and our findings support and emphasize the importance of this recommendation. Still, cefepime neurotoxicity can occur despite dose adjustments [19] and in our study, of those with cefepime neurotoxicity, 28.9% (four of fourteen patients with known doses) developed neurologic symptoms despite a standard dose reduction based on CL_Cr_. It is also notable that neurotoxicity can occur despite concurrent hemodialysis [20], though one expects the neurologic symptoms to improve after a few days if the dose has been appropriately adjusted and if flow rates are adequate. The current practice of cefepime dosing is dictated by standard algorithms according to CL_Cr_. Cefepime concentrations in the blood or cerebrospinal fluid have been performed rarely for research purposes and are not customarily performed in clinical practice. However, empirical dosing on the basis of one of these algorithms may not be sufficient to prevent accumulation of cefepime to ‘toxic’ concentrations in certain individuals because extreme pharmacokinetic deviations can occur while adhering to a standard algorithm [21]. In one prospective study of 20 ICU patients who received cefepime based on standard dose reductions according to CL_Cr_, plasma cefepime concentrations varied by two- to three-fold at peak levels and up to 40-fold at trough levels [21]. In addition, dosing medications for patients with CKD may be complicated because the calculated GFR or serum creatinine do not always correlate well with true renal function of these patients.

There are limitations to our study. This was not a prospective study of all patients in the ICU receiving cefepime, and thus we cannot estimate the true incidence of cefepime neurotoxicity in that population. While the modified Delphi method did mandate that there was no alternative cause of adverse neurologic symptoms, the retrospective nature of the study makes it impossible to know with full certainty that cefepime alone was the cause. Other variables could have potentially confounded this assessment and may have been in part responsible for symptoms that we ascribed to cefepime. However, in addition to the criteria mandating the exclusion of alternative causes, each potential case was reviewed independently by five clinicians to minimize this possibility.

In addition, EEGs were not performed in the majority of our patients, so it is possible we underestimated the occurrence of electrographic seizures or NCSE. Nevertheless, this may not have been clinically relevant because the patients in our series who did not have EEGs improved clinically with merely the discontinuation of cefepime and many patients described in the literature recover with simply discontinuing cefepime and without antiepileptic therapy [8,9,12,22]. In this series, the diagnosis of cefepime neurotoxicity was made by clinical assessment and consensus and we did not have blood or CSF levels of cefepime available. While diagnosis by clinical assessment more closely resembles current clinical practice, a prospective study in which cefepime levels are measured and correlated with clinical and electrophysiologic data would be useful to identify a therapeutic range that could potentially be used for drug monitoring in the future.

## Conclusions

Cefepime neurotoxicity affects critically ill patients with chronic kidney disease and causes encephalopathy, myoclonus, and less commonly seizures in ICU patients. It most commonly occurs when standard dose adjustments for renal function are not performed, but can occur despite dose adjustment in a minority of patients. Cefepime should be discontinued in ICU patients with renal failure who have encephalopathy if no alternative diagnosis is more likely, given that it is a potentially reversible cause of severe neurologic impairment.

## Key messages

•Among critically ill patients, cefepime neurotoxicity affects predominantly patients with pre-existent kidney disease.

•Encephalopathy and myoclonus are the most common clinical presentations of cefepime neurotoxicity in the ICU.

•Dose adjustments for renal function are critical to minimize the risk of cefepime neurotoxicity.

## Abbreviations

CKD: Chronic kidney disease; CLCr: Creatinine clearance; EEG: Electroencephalogram; eGFR: Estimated glomerular filtration rate; ICU: Intensive care unit; IQR: Interquartile range; NCSE: Nonconvulsive status epilepticus.

## Competing interests

The authors declare that they have no competing interests.

## Authors’ contributions

JF contributed to study design, data acquisition, data analysis, data interpretation, and drafting the manuscript. EK contributed to study design, data acquisition, and drafting the manuscript. SH contributed to data acquisition, and critically revising the manuscript. SC contributed to study design, and critically revising the manuscript. EW contributed to data acquisition, interpretation of data, critically revising the manuscript, and study supervision. AR contributed to study conception and design, data acquisition and interpretation, critically revising the manuscript, and study supervision. All authors have approved submission of this manuscript.
